# The effects of resistance training on muscle strength, joint pain, and hand function in individuals with hand osteoarthritis: a systematic review and meta-analysis

**DOI:** 10.1186/s13075-017-1348-3

**Published:** 2017-06-13

**Authors:** Nicoló Edoardo Magni, Peter John McNair, David Andrew Rice

**Affiliations:** 1 Health and Rehabilitation Research Institute, Auckland University of Technology, 90 Akoranga Drive, Northcote, Auckland, 0627 New Zealand; 2 Waitemata Pain Service, Department of Anaesthesiology and Perioperative Medicine, North Shore Hospital, Waitemata DHB, 124 Shakespeare Road, Westlake, Takapuna, Auckland, 0622 New Zealand

**Keywords:** Hand osteoarthritis, Rehabilitation, Conservative treatment, Resistance training, Muscle strength, Grip strength, Pain, Function

## Abstract

**Background:**

Hand osteoarthritis is a common condition characterised by joint pain and muscle weakness. These factors are thought to contribute to ongoing disability. Some evidence exists that resistance training decreases pain, improves muscle strength, and enhances function in people with knee and hip osteoarthritis. However, there is currently a lack of consensus regarding its effectiveness in people with hand osteoarthritis. Therefore, the aim of this systematic review and meta-analysis was to establish whether resistance training in people with hand osteoarthritis increases grip strength, decreases joint pain, and improves hand function.

**Methods:**

Seven databases were searched from 1975 until July 1, 2016. Randomised controlled trials were included. The Cochrane Risk of Bias Tool was used to assess studies’ methodological quality. The Grade of Recommendations Assessment, Development, and Evaluation system was adopted to rate overall quality of evidence. Suitable studies were pooled using a random-effects meta-analysis.

**Results:**

Five studies were included with a total of 350 participants. The majority of the training programs did not meet recommended intensity, frequency, or progression criteria for muscle strengthening. There was moderate-quality evidence that resistance training does not improve grip strength (mean difference = 1.35; 95% confidence interval (CI) = –0.84, 3.54; *I* ^2^ = 50%; *p* = 0.23 ). Low-quality evidence showed significant improvements in joint pain (standardised mean difference (SMD) = –0.23; 95% CI = –0.42, –0.04; *I* ^2^ = 0%; *p* = 0.02) which were not clinically relevant. Low-quality evidence demonstrated no improvements in hand function following resistance training (SMD = –0.1; 95% CI = –0.33, 0.13; *I* ^2^ = 28%; *p* = 0.39).

**Conclusion:**

There is no evidence that resistance training has a significant effect on grip strength or hand function in people with hand osteoarthritis. Low-quality evidence suggests it has a small, clinically unimportant pain-relieving effect. Future studies should investigate resistance training regimes with adequate intensity, frequency, and progressions to achieve gains in muscle strength.

**Electronic supplementary material:**

The online version of this article (doi:10.1186/s13075-017-1348-3) contains supplementary material, which is available to authorized users.

## Background

Hand osteoarthritis (OA) is present in 26% of females and 13% of males over the age of 71 [1]. Despite its relevance in terms of pain, disability, and economic burden on society, OA has often been referred to as ‘the forgotten disease’ [2]. Compared with the knee and hip joints, there are far fewer studies that have focused on conservative treatment for this pathology. Current clinical management of hand OA is centered on medications, which have been shown to be associated with notable side effects (e.g., ulcers, bleeding, renal failure, opioid addiction) [3]. The need for more effective and safe conservative interventions has been advocated by a number of authors [2, 4, 5]. Among the conservative treatments available for OA, exercises have been shown to be cost-effective and useful in improving quality of life [6]. Exercise aims to reduce the magnitude of change observed in strength, joint range of motion, proprioception, and alignment, which are often impaired due to the natural course of the disease and disuse [7]. Such impairments lead to reductions in function and quality of life [8].

Resistance training is an exercise intervention that has been utilised to decrease symptoms, impairment, and improve function in individuals with OA at the knee and hip [9, 10]. Several studies have demonstrated its effectiveness and this treatment modality is included in the American College of Rheumatology 2012 treatment guidelines for knee and hip OA, but not for hand OA [11]. The EULAR 2007 recommendations for the management of hand OA suggested the use of education plus exercise for the treatment of this pathology [12]. However, findings from only one randomised controlled trial (RCT) were used to support this recommendation and there was no direct evidence for education or exercises alone for the treatment of hand OA.

A number of studies have highlighted reduced muscle strength in those with hand OA [1, 13–16]. Furthermore, it is well known that many tasks of work and daily living require notable force to be exerted to complete them successfully [17]. Therefore, one might expect greater attention to have been paid to limiting muscle strength deficits through interventions such as resistance training. Previous reviews on hand OA have highlighted the limited number of studies assessing the effect of exercise on people with hand OA [4, 5, 18, 19]. To date, no reviews have focused specifically on the efficacy of resistance training exercises for hand OA and examined the training regimes adopted in the intervention studies. Thus, the aim of the current study was to perform a systematic review and meta-analysis of the effect of resistance training on grip strength, joint pain, and hand function in people with hand OA. Based on findings from studies in other joints affected by OA [9], we hypothesised that resistance training would improve muscle strength, joint pain, and function in people with hand OA.

## Methods

### Design and search strategy

This systematic review was conducted in accordance with the Preferred Reporting Items for Systematic Reviews and Meta-Analyses (PRISMA) guidelines [20]. The search strategy was based on the Population, Intervention, Comparison and Outcome (PICO) format. The electronic databases EBSCO host (CINAHL, MEDLINE, SPORTDiscus), Allied and Complementary Medicine Database (AMED) via OVID, Cochrane Central Register of Controlled Trials via Wiley, Web of Science, and Scopus were searched between 1975 and July 2016. The search was limited to published studies including human participants older than 18 years and published in English, Italian, or Spanish. The keywords utilised for the search included: hand(s), thumb(s), carpometacarpal(s), trapeziometacarpal(s), wrist(s), osteoarthr(itis)(osis)(itic), OA, train(ining)(ed), strength(ening)(ened), exercis(e)(ed)(es)(ing), physiotherap(y)(ist), physical therap(y)(ist), rehab(ilitation)(ilitative), manual therap(y)(ies), RCT(s), random(ly)(ised), trial(s)(led), experiment(s)(al). An additional table explains the search strategy in more detail (See Additional file 1: Table S1). Each database was searched by two people.

### Eligibility criteria

To be included in this review, studies must have been investigating the effects of resistance training in adults with hand OA. Eligible papers were published RCTs. Studies were considered if they included a between-group comparison after treatment in people with hand OA. Because this review was focused on the effect of resistance training, studies had to compare resistance training interventions with a nonexercising control intervention to be eligible for inclusion. Studies including multimodal intervention (e.g., splinting, manual therapy, ultrasound, yoga) were excluded. Studies including exercise without reference to resistance/strength training were not suitable for inclusion. The primary variables of interest were grip strength, joint pain, and hand function. Systematic, narrative reviews and experimental studies were identified and manual searches of their reference lists were undertaken to identify additional studies. Forward searches of included studies were completed in Google Scholar and Scopus.

### Study inclusion

All of the studies identified were collected in bibliographic software (Endnote X7; Thomson Reuters), where the inclusion and exclusion criteria were applied by two individuals. All duplicated studies were eliminated before title and abstract screening. The retained articles were retrieved in full text and assessed for inclusion. Disagreement on study inclusion was first discussed and if consensus was not reached the opinion of a third person was sought. A search of the reference lists of the included studies was undertaken to identify further articles.

### Risk of bias and overall quality of evidence

Using the risk of bias table suggested by the Cochrane Statistical Methods Group and the Cochrane Bias Methods Group [21], a critical appraisal of each study was performed by two researchers. The risk of bias table’s seven items assessed the internal validity of the studies. Each item was scored as low risk, high risk, or unclear risk.

To evaluate the overall quality of the evidence, the Grade of Recommendations Assessment, Development, and Evaluation (GRADE) system was utilised [22]. The quality of evidence was downgraded by one point from high quality for each factor that we encountered: risk of bias (if it was deemed that the bias may affect trial outcomes); inconsistency of results (wide variance of effect sizes or significant or large heterogeneity between trials: *p* < 0.05, *I* ^2^ > 50%); indirectness (application of intervention, intervention, or outcomes that differed from what we indicated in our PICO research question); and imprecision (optimal information size not met). A GRADE profile was completed for each pooled estimate. Two reviewers judged whether these factors were present for each outcome and in cases of disagreement a third reviewer was involved. The quality of evidence was defined as: high (the authors are confident that the true effect is close to the one estimated); moderate (the authors are moderately confident in the effect estimate); low (the true effect may be significantly different from the estimated); and very low (the true effect is most likely different from the estimated) [23].

### Data extraction

Descriptive statistics (means, standard deviations) for demographic and pre–post outcome dependent variables were extracted and cross-checked. When appropriate, the postintervention values for the exercise and control groups were used to calculate the mean difference (MD) or the standardised mean difference (SMD), which was the difference between groups values, divided by the pooled SD, with adjustment for small sample sizes (Hedges g: SMD). If more information was required for the quantitative analysis, authors were contacted to obtain further data.

### Data synthesis and analysis

Meta-analysis was performed in Review Manager (RevMan) software (version 5.3; Cochrane Collaboration) using the inverse variance method. We assumed that the studies’ variability, beyond subject-level sampling error, was random and consequently we adopted a random-effect model [20, 24]. Effect sizes of 0.2, 0.5, and 0.8 were considered small, medium, and large, respectively [25]. Publication bias was assessed by visually inspecting funnel plots [23]. Statistical heterogeneity was assessed using chi-square tests and the *I* ^2^ statistic, the latter providing a measure of the proportion of the observed variance that would remain if the sampling error was eliminated [26]. Where this proportion is of further interest, Borenstein et al. [26] have suggested that 95% prediction intervals should be calculated to appreciate the variability of the true effect size within the population under study.

## Results

**Fig. 1:**
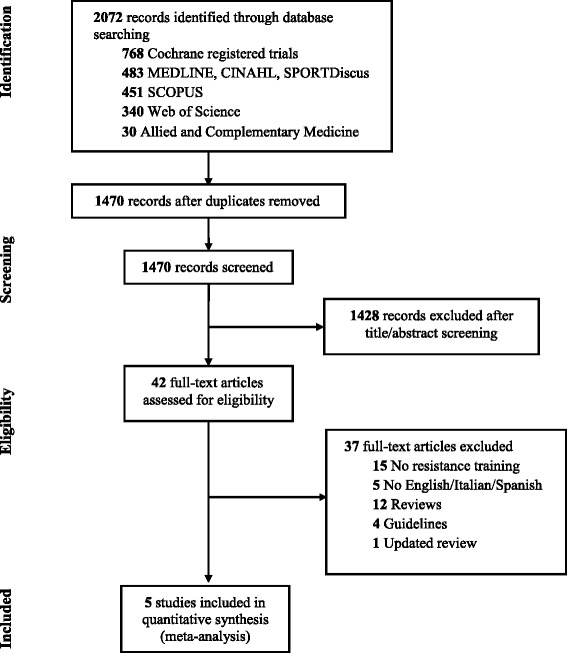
RCT selection throughout the review

The initial search identified 2072 papers. After duplicate elimination, 1470 studies underwent title and abstract screening, resulting in 42 studies considered suitable for inclusion. Following full paper review, five articles met the criteria for inclusion. Figure 1 outlines the RCTs selection through the review. No additional papers were retrieved from previous reviews, reference searches, or forward searches of included studies. Table 1 presents a comprehensive description of each trial included in the paper. A summary of findings and GRADE quality ratings are reported in Table 2.

**Table 1:** Characteristics of included studies and intervention

Study	Participants	Interventions	Outcome (follow-up time): statistical significance	Baseline differences
Dziedzic et al. (2015) [27]	*RGb* = 65 *CGb* = 65 *N* = 104 66% F 66 (9.1) years old	*RG* (*n* = 55): supervision = 1 group session/week (for 4 weeks). exercise = elastic bands fingers e/f, Play-Doh finger e/f (? % MVC), 0.5–0.75 kg wrist e/f dosage = 3 reps/day, every day progression = up to 10 reps/day *CG* (*n* = 49): leaflet and advice (extensive information)	Grip strength (24 wks): NS AUSCAN pain (12 wks): NS AUSCAN function (12 wks): NS	Strength (*p* = ?) Pain (*p* = 0.6) Function (*p* = 0.5)
Hennig et al. (2015) [31]	*RGb* = 40 *CGb* = 40 *N* = 71 100% F 60.8 (7) years old	*RG* (*n* = 37): supervision = 1 individual session with 8 follow-up calls exercise = elastic bands e/a thumb, rubber ball for grip strength (100% MVC) dosage = 10 reps (weeks 1 and 2), 3 days/week progression = 12 reps (weeks 3 and 4), 15 reps (weeks 5–12), 3 days/week *CG* (*n* = 34): leaflet and advice (limited information)	Grip strength (12 wks): S NRS pain (12 wks): S FIHOA (12 wks): S	Strength (*p* = 0.4) Pain (*p* = ?) Function (*p* = ?)
Lefler and Armstrong (2004) [28]	*RGb* = ? *CGb* = ? *N* = 19 90% F 81 (9) years old	*RG* (*n* = 9): supervised = every session (for 6 weeks); exercise = pinch grip lifting (isometric, 6-sec holds), wrist rolls (isotonic) (MVC = 40%) dosage = 10 reps, 3 days/week progression = up to 15 reps at 60% MVC isometric, 6–8 reps more than 60% MVC isotonic *CG* (*n* = 10): no intervention	Grip strength (6 wks): S Likert pain scale (6 wks): NS	Strength (*p* = 0.08) Pain (*p* = 0.53)
Østerås et al. (2014) [29]	*RGb* = 65 *CGb* = 65 *N* = 120 90% F 66 (9) years old	*RG* (*n* = 57): supervised = 4 group sessions (weeks 1–3 and 8) exercise = shoulder e/f, biceps curl, elastic band e/a thumb, pipe squeeze (100% MVC) dosage = 10 reps, moderate/vigorous intensity (weeks 1 and 2), 3 days/week progression = 15 reps (weeks 3–12) *CG* (*n* = 63): usual care (GP visit)	Grip strength (12 wks): NS NRS pain (12 wks): S FIHOA (12 wks): NS	Strength (*p* = 0.3) Pain (*p* = 0.4) Function (*p* = 0.26)
Rogers and Wilder (2009)^a^ [30]	*RGb* = 76 *CGb* = 76 *N* = 46 87% F 75 (6.7) years old	*RG* (*n* = 46): supervised = 1 individual session exercise = gripping (16–19% MVC), key pinch, fingertip pinch all with rubber ball dosage = 10 reps (weeks 1, 2, 3 and 4), every day progression = 12 reps, 15 reps, 20 reps all increased every fourth week *CG* (*n* = 46): sham hand moisturiser	Grip strength (16 wks): NS AUSCAN pain (16 wks): NS AUSCAN function (16 wks): NS	Strength (*p* = 0.96) Pain (*p* = 0.84) Function (*p* = 0.87)

*RGb* participants allocated to the resistance training group, *CGb* participants allocated to the control group, *N* participants retained at follow-up, *F* female, *RG* resistance training group, *n* group sample size retained at follow-up, *wks* weeks, *e/f* extension/flexion, *MVC* maximum voluntary contraction, *?* unable to calculate/unknown, *reps* repetitions, *CG* control group, *AUSCAN* Australian Canadian Osteoarthritis Hand Index, *NS* nonsignificant, *e/a* extension/abduction, *NRS* Numerical Rating Scale, *FIHOA* Functional Index of Hand Osteoarthritis, *S* significant

^a^Cross-over study design

**Table 2:** Summary of findings: resistance training compared with no exercise for hand osteoarthritis

Outcomes	Anticipated absolute effects^*^ (95% CI)		Number of participants (studies)	Quality of evidence (GRADE)	Comments
	Risk with no exercise	Risk with resistance training			
Grip strength (at study completion) assessed with: hand dynamometer. Follow-up: range 6–24 weeks	Mean grip strength (at study completion) in the control group was 17.7 kg	Mean grip strength (at study completion) in the intervention group was 1.35 kg higher (0.84 lower to 3.54 higher)	350 (5 RCTs)	⨁⨁⨁◯ moderate^a^	MD 1.35 kg (95% CI = –0.84, 3.54). Relative increase 8% with resistance exercise (95% CI = –5% weaker, 20% stronger). MCID for grip strength is 20%^b^
Hand pain (at study completion) assessed with: AUSCAN pain, 11-point NRS, Likert scale. Lower scores mean less pain. Follow-up: range 6–16 weeks	Pain score in the resistance training groups was on average *–0.23 SDs* (–0.42 lower to –0.04 lower) *lower* than in the control groups.^e^		379 (5 RCTs)	⨁⨁◯◯ low^a,c^	These results can be interpreted as an improvement of 0.46 (95% CI = 0.08, 0.84) points on a 11-point NRS scale.^d^ MCID for pain is 2 points [39]
Hand function (at study completion) assessed with: AUSCAN function, FIHOA. Lower scores mean better function. Follow-up: range 6–16 weeks	The function score in the resistance training groups was on average –0.10 SDs (–0.33 lower to 0.13 higher) lower than in the control groups.		363 (4 RCTs)	⨁⨁◯◯ low^a,c^	As a rule of thumb, 0.2 SDs represents a small difference, 0.5 a moderate difference, and 0.8 a large difference

**Patient or population:** hand osteoarthritis

**Setting:** general practice, community, retirement villages

**Intervention:** resistance training

**Comparison:** no exercise

*CI* confidence interval, *AUSCAN* Australian Canadian Osteoarthritis Hand Index, *NRS* Numerical Rating Scale, *FIHOA* Functional Index of Hand Osteoarthritis, *RCT* randomised controlled trial, *MD* mean difference, *SD* standard deviation, *MCID* minimal clinically important difference, *GRADE* Grade of Recommendations Assessment, Development, and Evaluation

^*^ Risk in the intervention group (and its 95% CI) is based on the assumed risk in the comparison group and the relative effect of the intervention (and its 95% CI)

**GRADE Working Group grades of evidence**

**High quality** **: **We are very confident that the true effect lies close to that of the estimate of the effect

**Moderate quality:** We are moderately confident in the effect estimate (the true effect is likely to be close to the estimate of the effect, but there is a possibility that it is substantially different)

**Low quality:** Our confidence in the effect estimate is limited (the true effect may be substantially different from the estimate of the effect)

**Very low quality:** We have very little confidence in the effect estimate (the true effect is likely to be substantially different from the estimate of effect)

^a^ Downgraded because few participants (imprecision)

^b^ MCID for grip strength in people following a radial fracture [47]

^c^ Downgraded because participants were not blinded to intervention (risk of bias)

^d^ The control group pain mean (SD) 4.6 (2) was calculated by averaging the 11-point NRS scores of Dziedzic et al. [27], Hennig et al. [31], and Østerås et al. [29]^e^ This result was statistically significant (*p* = 0.02)

### Study characteristics

The participants’ count was based on the participants retained at the follow-up period (see Table 1). Out of the 350 participants, 305 (87%) were female. Mean age ranged from 61 to 81 years old. The primary outcome measures were grouped into grip strength, joint pain, and self-reported hand function. Grip strength was assessed through a dynamometer [27–31]. Joint pain measurements included the AUSCAN pain subscale [30], the Numerical Rating Scale (NRS) [27, 29, 31], and a six-point Likert scale [28]. Self-reported measures of hand function included the AUSCAN function subscale [27, 30] and the Functional Index of Hand Osteoarthritis (FIHOA) [29, 31].

### Experimental intervention

#### Duration and supervision

Dziedzic et al. [27] had an ongoing exercise program with no set ending date. The remaining studies adopted training programs of 6–16 weeks [28–31]. Outcome measures were assessed at the end of the exercise period, except Dziedzic et al. [27] who measured grip strength at 24 weeks after participants’ inclusion in the trial. Two studies supervised participants individually over one session, followed by a home exercise program (HEP) [30, 31]. Two studies supervised participants over four group sessions [27, 29]. Østerås et al. [29] provided group sessions over the first 3 weeks and towards the end of the trial (week 8). The timing of participant attendance in the group sessions of the study by Dziedzic et al. [27] was not clear. Lefler and Armstrong [28] reported that participants were supervised over 6 weeks, three times a week (18 sessions). However, it is not clear whether the sessions were individual or group sessions.

#### Training modality and frequency

Gripping and forearm flexor exercises were performed in all studies through different exercises (see Table 1). Three studies included specific exercises to improve thumb extension and abduction strength [27, 29, 31]. Finger and wrist extensor strengthening exercises were performed by two studies [27, 28]. Shoulder strengthening exercises were performed in only one study [29]. Two studies required participants to exercise every day [27, 30] and three studies to exercise three times per week [28, 29, 31]. Repetitions at the beginning of training for each exercise ranged from three [27] to 10 [28–31].

#### Exercise intensity and progression

Only one study reported the percent of maximum voluntary contraction (40% of MVC) at which participants exercised [28]. Three other studies [29–31] presented enough data to infer an exercise load. Hennig et al. [31] and Østerås et al. [29] reported that participants were asked to ‘squeeze as hard as possible’ (100% of MVC) while performing gripping exercises. Rogers and Wilder [30] had participants perform exercises between 16 and 19% of MVC. We were unable to calculate the exercise intensity for Dziedzic et al. [27] because there was not enough information available. All studies progressed the exercises by increasing the number of repetitions up to a maximum of 20. Only one study [28] included a progressive increase in exercise load (up to 60% of MVC).

### Control intervention

Two studies provided the control group with a leaflet and advice over one session [27, 31]. Two studies did not provide any intervention to the control group [28, 29]. Østerås et al.’s [29] control group was allowed to receive usual care, which in Norway consisted of general practitioner visits only. Rogers and Wilder [30] crossed over the same participants from a placebo hand moisturiser to the resistance training group and vice versa, with a 16-week washout period.

### Risk of bias

**Fig. 2:**
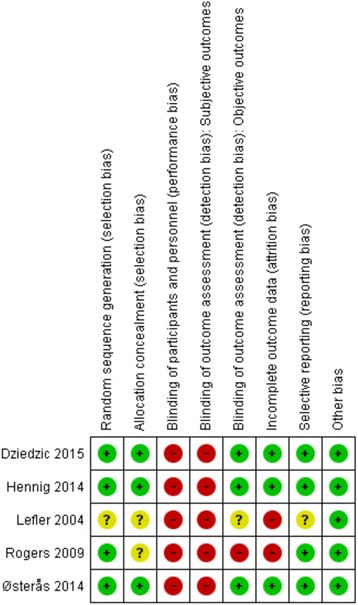
Risk of bias summary across studies

The risk of bias across the studies varied substantially (see Fig. 2). All of the studies failed to blind the treatment providers and participants due to the nature of the intervention. Dziedzic et al. [27], Hennig et al. [31], and Østerås et al. [29] presented the lowest risk of bias. Rogers and Wilder [30] and Lefler and Armstrong [28] presented the highest risk of bias.

### Overall quality of evidence and meta-analyses

**Fig. 3:**
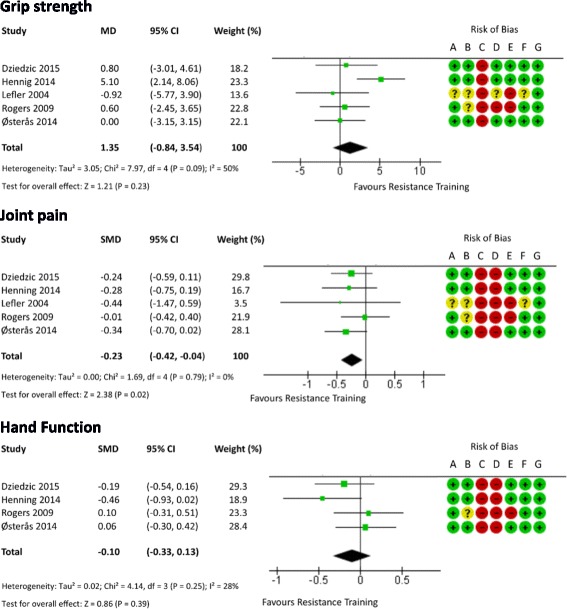
Forest plot showing the effect of resistance training on grip strength, pain, and function in people with hand OA. *CI* confidence interval, *MD* mean difference, *SMD* standardised mean difference

**Fig. 4:**
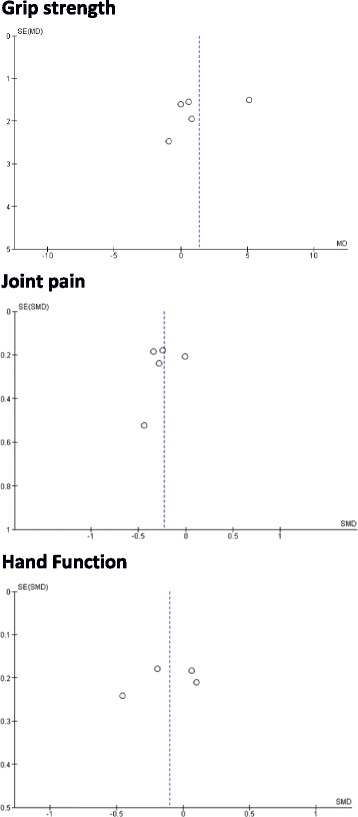
Funnel plot for grip strength, pain, and function in people with hand OA. *MD* mean difference, *SE* standard error, *SMD* standardised mean difference

The results from the meta-analyses for grip strength, joint pain, and hand function are presented as forest plots in Fig. 3. Funnel plots for each outcome are provided in Fig. 4. Visual inspection did not reveal publication bias.

#### Grip strength

Out of the five studies included, only two studies showed a significant change in grip strength after resistance training compared with the control group [28, 31]. The pooled results provide moderate-quality evidence that resistance exercises, as performed by these combined interventions, do not improve grip strength (MD 1.35 (95% CI = –0.84, 3.54), *p* = 0.23). The *I* ^2^ value was 50% (χ^2^ = 7.97, *p* = 0.09). The prediction interval indicated that 95% of the effect of resistance training would lie between –5.2 and 7.9 kg.

#### Joint pain

Most of the studies included in the present review showed a trend toward improvement in pain intensity for the resistance training group. However, only two studies reported statistically significant changes in pain [29, 31] compared with the control group. The pooled results provide low-quality evidence that resistance exercises provide pain relief (SMD –0.23 (95% CI = –0.42, –0.04), *p* = 0.02). The *I* ^2^ value was 0% (χ^2^ = 1.69, *p* = 0.79). The prediction interval indicated that 95% of effect sizes would lie between –0.54 and 0.08.

#### Hand function

Only one study reported significant differences in self-reported hand function after resistance training compared with the control group [31]. The pooled results provide low-quality evidence that resistance exercises do not improve hand function (SMD –0.1 (95% CI = –0.33, 0.13), *p* = 0.39). The *I* ^2^ value was 28% (χ^2^ = 4.14, *p* = 0.25). The prediction interval indicated that 95% of effect sizes would lie between –0.9 and 0.7.

## Discussion

This meta-analysis assessed the effect of resistance training on grip strength, joint pain, and hand function in participants with hand OA. It was clear that there are very few experimental studies which have specifically addressed the effects of resistance training in this population. Previous reviews have highlighted this problem, and also emphasised the general scarcity of research involving conservative interventions for hand OA [2, 4, 5, 17, 18, 32]. These findings are surprising considering that resistance training has been used in other forms of OA with positive effects on pain, function, and patients’ quality of life [7]. The five studies included had small sample sizes and the outcome data were not available for participants lost at follow-up.

There was ‘moderate-quality evidence’ that the resistance training utilised in the included studies did not improve grip strength. Of note, our overall finding concerning grip strength is in contrast to a recent review by Østerås et al. [32]. These authors noted that there was a strong trend for an improvement following training. This discrepancy most likely is related to the data analysed in the meta-analysis; that is, Østerås et al. [32] included findings from an abstract in their analysis, and furthermore, they were not able to include additional data concerning the findings of Rogers and Wilder’s [30] work (which we were able to include after personal communication).

Nevertheless, our findings are surprising because all studies included in our analysis included gripping or forearm flexor exercises against resistance. The absence of grip strength improvement in the majority of the studies raises some questions regarding the appropriateness of the resistance training programs utilised. In addition, the technique used in the measurement of grip strength may not be congruent with the types of exercise undertaken in the intervention [33]. For instance, in the current review only two papers identified the hand position utilised for grip strength testing [29, 30], and in both instances the same position was utilised for all participants. This would limit the observation of strength gains if individuals trained at muscle lengths shorter or longer than the testing position (training specificity principle) [34].

Additionally, a key point in resistance training guidelines concerns the volume of exercise required. The majority of the studies adopted exercise frequency, intensity, sets, repetitions, and progression which are not sufficient to induce strength gains in older adults [35]. For instance, it was apparent that four studies progressed participants by increasing the number of repetitions rather than the exercise intensity [27, 29–31], and were therefore pursuing an approach that is more efficacious for enhancing muscle endurance as compared with strength [35]. With regard to absolute exercise intensity, it has been recommended that loads of at least 60% of MVC are utilised with intensity increasing as training progresses to levels approaching 80% of MVC [35]. Only two studies [29, 31] reported resistance training loads sufficient to induce increases in muscle strength (100% of MVC). Of these, Hennig et al. [31] reported significant changes in grip strength while Østerås et al. [29] reported only limited changes. In both cases, participants were instructed to squeeze an object as hard as possible. Because grip forces are unable to be measured using such a protocol, there is no way of being sure that participants were indeed working at 100% of MVC, as compared with exercising at resistance levels that can be quantified more accurately (e.g., on a hand-held dynamometer or weights).

Pain during exercise may have influenced load and intensity performed. In this regard, Hennig et al. [31] reported that participants’ joint pain intensity immediately post exercise was high (NRS: 5.6 ± 2.2) while no data were available for the study by Østerås et al. [29]. It is possible that in the study by Østerås et al. [29], in which strength changes were small, participants self-limited the exercise intensity to avoid increases in joint pain. Similarly, the low exercise load utilised by the other included studies [28, 30] may reflect the intention to avoid high joint compressive forces and further damage to the articular cartilage. However, there is a growing body of evidence suggesting that high levels of pain during or immediately after resistance training sessions (up to 6 on a NRS scale) do not negatively affect outcomes, but rather improve overall levels of pain for the duration of the training program in people with hand and knee OA [31, 36, 37]. Such pain intensities have been previously considered acceptable in people with OA, on the condition that pain intensity returns to baseline values within 24 hours of the previous session [31, 38].

There was low-quality evidence suggesting that resistance training reduces joint pain. Additionally, when the standardised mean difference calculated in the current study was transformed into absolute values on a 11-point NRS scale (see Table 2), the difference between groups was 0.46 points (95% CI = 0.08, 0.84), which does not reach the minimal clinically important difference of two points commonly used in OA trials [39]. At the knee joint, findings are more encouraging, with a RCT [37] reporting a mean reduction in pain of 2.3 points following high-intensity resistance exercises. There is no reason to suspect that such findings might not be possible at the hand given the mechanisms advanced for its success. These include muscle strengthening altering alignment and hence loading on damaged structures within a joint, reducing the potential for inflammation and hence pain. Other authors [40] have suggested that increased proprioceptive awareness leads to improved placement of joints during motion, reducing load. There is also a strong potential for an antinociceptive effect of resistance training through modulation of endogenous analgesia [41–43] and/or anti-inflammatory effects that may reduce peripheral and central sensitisation [44].

Low-quality evidence demonstrated that hand function was not improved following resistance training. Similar results were obtained by a recent review by Bertozzi et al. [18] which showed no significant effects of exercise interventions on hand function in people with thumb carpo-metacarpal joint OA. In contrast, Østerås et al. [32] found a trend (*p* = 0.07) toward exercise being beneficial for function. A number of factors may be associated with these findings. These include the assessment of function by questionnaires that do not include tasks that the participants find difficult to perform, questionnaires that focus primarily on tasks requiring fine motor control tasks, rather than strength tasks, and/or resistance training programs not targeting appropriate muscle groups. As suggested by van Baar et al. [10] and adopted by Hoeksma et al. [45], it may be that targeting the individual’s specific needs is a solution. However, where researchers take this pathway, it is important that they provide descriptions of the criteria which lead them to focus on a specific type of exercise, and also provide the training parameters and improvements that occurred for those participants. Without such information, readers have no way of discerning how to prioritise types of exercise that would be most valuable for their patients. In future studies, the resistance training exercises utilised could be described in detail according to the Consensus on Exercise Reporting Template (CERT) [46].

It may be viewed as a limitation of the current study that we chose to focus on studies utilising resistance training exercises only. We are aware that in clinical practice multimodal therapies are often utilised and a combination of conservative and pharmacological interventions are adopted. However, to optimise both the efficiency and cost-effectiveness of OA treatment it is important to understand which component(s) of an intervention offer the most benefit (or otherwise). Our focus on resistance training is also justified by the established effectiveness of this intervention in other joints such as knee OA [9]. Furthermore, a number of functional tasks at the hand require notable muscle forces to be generated and it has been suggested that 20–25 kg of grip strength is required for daily life activities [17]. An additional limitation of the present study is that a review protocol was not published before starting the search. We are aware that this is suggested by the PRISMA guideline. However, we prespecified the use of meta-analysis for all the outcomes chosen, which included strength, pain, and function. Another limitation is the small number of participants included in the meta-analysis. This was acknowledged and the overall quality of evidence was downgraded (see Table 2). Nevertheless, all of the studies except that by Lefler and Armstrong [28] performed power calculations, suggesting that the optimal information size was probably met. A per-protocol analysis was performed on the postintervention data reported in each study. Data for participants who dropped out were not available. Formal statistical analyses to assess publication bias were not performed due to the limited number of studies available. Visual inspection of funnel plots did not identify any clear indication of publication bias. In addition, the absence of clinically significant improvements in the main outcome variables makes the effects of any publication bias unlikely to change the main conclusions of our review. Finally, we need to acknowledge as a limitation the inclusion of studies published only in English, Spanish, or Italian.

## Conclusions

There is no evidence indicating that resistance training increases grip strength or has a clinically significant benefit on hand OA pain and function. However, this may be related to the paucity of studies and low-quality study designs. Future studies should consider focusing exercise programs specifically on identified muscle deficits as well as optimising exercise training parameters to achieve clinically significant strength improvements in people with hand OA.

## Additional file

Additional file 1: Table S1. Presenting the search strategy across different databases: keyword combination utilised across database searches. (DOCX 14 kb)

## Abbreviations

AUSCAN: Australian Canadian Osteoarthritis Hand Index
CI: Confidence interval
CERT: Consensus on Exercise Reporting Template
FIHOA: Functional index of hand OA
GRADE: Grade of Recommendations Assessment, Development, and Evaluation
HEP: Home exercise program
MCID: Minimal clinically important difference
MD: mean difference
MVC: Maximum voluntary contraction
NRS: Numerical Rating Scale
OA: Osteoarthritis
PICO: Population, Intervention, Comparison and Outcome
PRISMA: Preferred Reporting Items for Systematic Reviews and Meta-Analyses
RCT: Randomised controlled trial
SD: standard deviation
SMD: Standardised mean difference
