# Supplementary material for: The effects of resistance training on muscle strength, joint pain, and hand function in individuals with hand osteoarthritis: a systematic review and meta-analysis
Source: Arthritis Res Ther. 2017 Jun 13;19:131. doi: 10.1186/s13075-017-1348-3 (PMC5470180; doi:10.1186/s13075-017-1348-3)
Supplement: Additional file 1: Table S1. — Presenting the search strategy across different databases: keyword combination utilised across database searches. (DOCX 14 kb) [file 13075_2017_1348_MOESM1_ESM.docx]

| Additional File 1. Search strategy across different databases | |
| --- | --- |
| **Database searched** | **Search terms** |
| Cochrane registered trials via Wiley | (hand* OR thumb* OR carpometacarpal* OR trapeziometacarpa* OR wrist*)  AND  (osteoarthr* OR OA)  AND  (train* OR strength* OR exercis* OR physiotherap* OR "physical therap*" OR rehab*)  AND  (RCT* OR random* OR trial* OR experiment*) |
|  |  |
| EBSCO Host (MEDLINE, CINAHL, SPORTDiscus) | (hand* OR thumb* OR carpometacarpal* OR trapeziometacarpa* OR wrist*)  AND  (osteoarthr* OR OA)  AND  (train* OR strength* OR exercis* OR physiotherap* OR "physical therap*" OR rehab*)  AND  (RCT* OR random* OR trial* OR experiment*) |
|  |  |
| SCOPUS | (hand* OR thumb* OR carpometacarpal* OR trapeziometacarpa* OR wrist*)  AND  (osteoarthr* OR OA)  AND  (train* OR strength* OR exercis* OR physiotherap* OR "physical therap*" OR rehab*)  AND  (RCT* OR random* OR trial* OR experiment*) |
|  |  |
| Web of Science | (hand* OR thumb* OR carpometacarpal* OR trapeziometacarpa* OR wrist*)  AND  (osteoarthr* OR OA)  AND  (train* OR strength* OR exercis* OR physiotherap* OR "physical therap*" OR rehab*)  AND  (RCT* OR random* OR trial* OR experiment*) |
|  |  |
| Allied and Complementary Medicine via OVID | (hand* OR thumb* OR carpometacarpal* OR trapeziometacarpa* OR wrist*)  AND  (osteoarthr* OR OA)  AND  (train* OR strength* OR exercis* OR physiotherap* OR "physical therap*" OR rehab*)  AND  (RCT* OR random* OR trial* OR experiment*) |

A keyword search was utilised instead of database-specific subject heading searches due to the wide range of databases used, making the search strategy directly equivalent across the sources used. Additionally, the broader approach of keyword searching was a strengths of this review, providing comprehensive and extensive results as suggested by the Cochrane Handbook in section 6.4.4 (1).

References

[1] Higgins JPT, Green S. Cochrane handbook for systematic reviews of interventions version 5.1.0. 2011. Available from: [www.handbook.cochrane.org](http://www.handbook.cochrane.org)
